# News and Notes

**Published:** 1906-06

**Authors:** 


### NEWS AND NOTES.

DR. E. C. RIPLEY has recovered from a recent operation for appendicitis.

PLACARDS are to be placed on the houses inhabited by tuberculous patients in Oil City, Pa.

THE first building to be completed at the Jamestown Exposition will be the Pocahontas Hospital.

ON April 4th, Governor Stokes, of New Jersey, signed the bill substituting electrocution for hanging after March 1, 1906.

THE failure of the Legislature to appropriate $50,000 to the Johns Hopkins University will delay indefinitely its removal to the new suburban site.

THIRTY-TWO young men and one young woman were graduated from The Medical Department of Grant University in Chattanooga, Tenn., April 30.

AMERICAN MEDICINE, in its issue for March 31st, announced that in April that journal will suspend publication as a weekly, and will thereafter appear only twelve times a year.

PROF. AUGUST BIER, of Bonn, whose method of treating inflammations, not only acute, but chronic, by inducing hyperæmia, was commented on in our issue for February 24th, has been awarded the Kussmaul prize by the medical faculty of Heidelberg.

JOHN H. HENNIES, Ph.G., M.D., was graduated from the International Medical Missionary College, April the 5th. Dr. Hennies is the first to receive a diploma from this college.

---

DR. W. W. KEEN announces that he will close his private hospital permanently after June 1. Thereafter his patients will be taken care of in the Jefferson Medical College Hospital.

---

A BILL has been introduced into the Senate of New Jersey appropriating $70,000 per annum for five years for the purpose of exterminating the mosquitoes in the New Jersey marshes.

---

DR. L. S. MCMURTRY, of Louisville, president of the American Medical Association, was the guest of honor at the annual meeting of the Louisville Academy of Medicine, March 7, 1906.

---

THE Pennsylvania State Pharmaceutical Board intends to bring action against certain druggists in Philadelphia for intrusting the compounding of prescriptions to clerks without the proper State license.

---

NOTICE.—No doubt a great many physicians will be glad to learn that Scott & Bowne are no longer members of the Proprietary Association of America, having resigned from it several months ago.

---

THE North Idaho Medical Society will hold its next meeting at Coeur d'Alene City, Idaho, June 19th, and will be followed by a half-day spent at the handsome new St. Luke's Hospital, a Protestant institution in Spokane.

---

ON April 10th Senator Saxe's bill to establish a seaside park for the convalescent poor of New York city, was passed by the

Assembly. The bill, which now goes to the Mayor, authorizes the city to spend $2,500,000 on the project.

---

PROFESSOR CZERNY, who has held the chair of surgery in the University of Heidelberg since 1877, has resigned, in order to devote his entire time to the duties of director of the Institute of Cancer Research.

---

THE Cincinnati Board of Health, on the recommendation of the city health officer, has passed a resolution ordering the lodging-houses on the river front to discontinue the five-cent rooms, which are reported to be unsanitary in the highest degree.

---

THE Hungarian faster, Sacco, has recently finished a fast of forty-five days. He lost 55 pounds, but was in good physical condition at the end of the test. During the period he smoked 952 cigarettes and drank 22 bottles of table water.

---

THE Iowa State Board of Health has adopted a rule excluding tuberculous children and tuberculous teachers from the public schools of that State. Foolish legislation such as this would stop the antituberculosis crusade if it could be stopped.

---

THE engagement of Dr. Lewis M. Gaines, of Wake Forest College, N. C., to Miss Virginia Ethel Alexander, has been announced. The wedding will occur on the evening of the 12th of June at the home of the bride's parents, 257 Washington street, Atlanta, Ga.

---

IN the May issue of the *Johns Hopkins Hospital Bulletin,* Dr. Harvey Cushing gives an interesting description of the course of Operative Medicine in the Hunterian Laboratory, which has recently been completed and now affords the student an opportunity for practical work upon living animals.

---

IT has been decided at Washington that the crews of vessels plying between Southern ports of the United States and Havana need not be yellow fever immunes. This ruling will be a great advantage to the shipping companies in both countries.

---

A PROCESS of photographing germs by means of the so-called ultra violet rays of the spectrum has been evolved by Professor Ernst, of the Harvard Medical School. By this means, it seems, each germ "stands out separate." Heretofore the process of discovering the size and shape of germs has been carried on by chemical means.

---

DOM FOURNIER, a Benedictine monk of the Abbey of Solesmo, who before entering the order was a medical practitioner, has compiled a book on the canonized saints who have been members of the medical profession. He has succeeded in unearthing no fewer than sixty-eight. Most of them belong to the early days of Christianity.

---

SUPERVISING SURGEON-GENERAL WALTER WYMAN, of the Public Health and Marine Hospital Service, has called a conference, to consist of one delegate each from the health department of Mississippi, Louisiana, Alabama and Texas, to meet at Washington on Monday, April 16th, for the purpose of discussing quarantine matters.

---

IN England there exists much more antagonism in regard to vivisection than in this country. To such an extent does this feeling prevail that there is soon to be presented to the British

Parliament a petition seven miles long against the vivisection of dogs. It seems too bad that so much good paper should be wasted in such a manner.—*Med. Age.*

---

THE Supreme Court of Arkansas has declared as valid the State law passed in 1904, prohibiting the drumming of patients for doctors. It is held that the Visitors' Protective Association, of Hot Springs, was engaged in a lawful undertaking in endeavoring to have this law enforced, and should not have been enjoined from so doing by the lower court.

---

THE $25,000 appropriated by the Legislature to the Endowood Hospital for Consumptives, in the suburbs of Baltimore, will permit the erection of additional buildings, the laying out of the 75 acres of grounds, and the establishment of a country sanitarium. The benefits also can be extended to the poor. Private charity is expected to add $50,000 or more to the aid given by the State.

---

A DEATH occurred on February 20th, in the dentist's chair, under ethyl chloride, making, according to the *Lancet* for March 3d, which comments at length on the occurrence, the ninth on record. The *Lancet* quotes Prof. H. C. Wood's opinion, that ethyl chloride exerts a strong depressant effect on the heart, and deprecates its use in the aged and in those with embarrassed respiration.

---

THE Medical Society of the State of New York is taking a vote by mail as to whether "The Principle of Medical Ethics of the American Medical Association, being suggestive and advisory, shall be the guide of members in their relation to each other and to the public." The polls will be closed at the expiration of ten days after the submission of the question, which occurred April 27.

A STUDENT at the Creighton Medical College, Omaha, is about to sue the faculty for $50,000, on the ground that as the result of hazing he has been completely incapacitated. He states that he was dragged from his classroom by Sophomores, who intended to throw him into a ventilating shaft. He fought, and was kicked in the back, his spine being injured, and he has had to use crutches ever since.

---

THE Alumni Association of the Medical College of the State of South Carolina, Charleston, held its annual meeting April 11, at which the following officers were elected: Dr. Robert S. Cathcart, Charleston, president; Drs. T. H. Tuten, Crockettsville; J. R. Bell, Due West; Henry Horlbeck, Columbia, and Arthur F. Doty, Sumter, vice-president, and Dr. C. Bunting Colson, Charleston, secretary and treasurer.

---

DR. A. E. WRIGHT, formerly demonstrator of pathology at the University of Cambridge, demonstrator of physiology at the University of Sydney, N. S. W., and professor of pathology at the Army Medical School at Netley, and now lecturer on pathology at St. Mary's Hospital, London, the discoverer of the opsonic index, has been recommended by the council of the Royal Society of London for election as a fellow of the society.

---

THE Medical Club of Budapest has voted to have an artistic, tall drinking-vessel made as a memorial to Semmelweis, to be used only on great occasions, when the "pokal" will be handed around with great ceremony. It will be used first at a banquet to the memory of Semmelweis, on his anniversary; and in the following years, only when some important medical or scientific question is to be discussed or tribute paid to some departed notability.

THE Greenville County Medical Association scored the first victory in its campaign against illegal practitioners January 29, when "Dr." C. P. Price was convicted of the unlawful practice of medicine. The prisoner is said to have fled the country. The law provides for a fine of not less than $50 nor more than $300, or imprisonment of not less than thirty nor more than ninety days, or both, at the discretion of the trial judge.

---

ACCORDING to an article in the *Progrès Médical* for April 7, Belgium is the best provided with official laboratories for bacteriologic investigation of any country in the world to date. Each province has one or more, and the number of specimens sent for examination is constantly increasing every year. Since its foundation in 1896 to 1905, the laboratory at Liège has made about 25,000 analyses. Of this number 14,045 were made during the year 1904.

---

How to dust a room is explained in a card issued by the New York Charity Organization Society's Committee on the Prevention of Tuberculosis. One should not use a feather duster, because this does not remove the dust from the room, but only brushes it into the air so that it is inhaled; or the dust settles, and the work must be done over again. Soft, dry cloths should be used, which are shaken frequently out of the window; or slightly moistened cloths, which may be rinsed out in water after using them.

---

A NURSE at the University Clinic at Bonn gave a caustic powder by mistake to a patient who had entered the clinic for an operation the next day. The action of the caustic caused injuries which will persist to a certain extent, and the patient sued the university for $3,000. The university claimed that it was a State institution and not individually responsible, but the local and supreme courts decided that the university was legally responsible for injury resulting from carelessness in its clinic and sustained the claim for damages.

---

A BILL has just been introduced into the British Parliament designed to carry out the unanimous recommendation of the physical deterioration bill: 1, To probibit the sale of tobacco and cigarettes to children below a certain age; 2, to prohibit the sale of tobacco and cigarettes in candy or other shops frequented by children. The operative clause would enact that any person selling, giving or supplying tobacco in any form to or for the use of any person under the age of sixteen would be liable to a fine on first conviction not exceeding $5, and on further conviction not exceeding $10.

---

THE Davidson (N. C.) Medical College, heretofore an adjunct of the Davidson College, is to be moved to Charlotte, N. C., and established as an independent institution. The trustees of the college have purchased a large lot in the center of the city, and work will shortly begin on the erection of a three-story building, in which the college will be domiciled. It is expected that the building will be ready for the opening of the college as an independent institution, next autumn. The trustees have not revealed their plans for the future beyond what is stated. The announcement is considered important, as it will be the State's first independent medical college.

---

A RECEPTION to Professor Friedrich Trendelenburg, of Leipsic, was given by Dr. Willy Meyer, at Delmonico's, on Friday evening, April 27, 1906. Professor Trendelenburg has come to this country on the invitation of the American Medical Association to attend the Boston meeting, prior to which he will visit Philadelphia, Baltimore and Washington; thence he goes west to Chicago, and to visit the Mayo's at Rochester, Minn. Professor Trendelenburg expects to attend the meeting of the American Surgical Association, which will be held at Cleveland on May 30. During his stay in New York he is the guest of Dr. Willy Meyer.

---

A DEPARTMENT of tropical diseases and hygiene has been established in connection with the Berlin Institute of Infectious Diseases. Dr. Claus Schilling has been appointed head of the new department. He took his doctor's degree at Munich in 1895, and was afterward assistant in the University Pathological Institute under Professor Bollinger. Later Dr. Schilling accepted a post in the Bacteriological Laboratory of the Imperial Health Bureau at Berlin. In 1900 he went out as government medical officer to Togo. For a year past he has been working in the Berlin Institute of Infectious Diseases. Dr. Schilling is the author of many contributions to bacteriological and pathological literature.

---

THE Rockefeller Institute for Medical Research was formally dedicated May 11th in New York City. There were present many men distinguished in every line of medical research. Addresses were delivered by President Charles W. Elliott, of Harvard; Dr. Nicholas Murray Butler, of Columbia; Dr. Emmett Holt and Dr. Wm. H. Welsh, of Baltimore. This institution, the most pretentious and best endowed of its kind in the United States, is the gift of John D. Rockefeller. The cost of the building was about $600,000. It is the intention to establish a hospital on the grounds of the institute in order to apply the latest discoveries in science to problems connected with the prevention and cure of disease.

---

THE COUNTRY DOCTOR OBSOLETE: The country doctor is rapidly becoming extinct as a species. The men one meets at these societies look, dress, talk and act, as the men do at any meeting of city physicians. The papers presented are quite up to the city standard, the discussions markedly above those of the city men. Therapeutics is discussed intelligently, scientifically, without undue optimism, without a trace of the silly pessimism too often assumed by the city physician to disguise his crass ignorance. The surgical experiences related would astonish some men who think the city clinics and clinicians do all of this work, or at least all that is well done.—*American Journal of Clinical Medicine.*

---

ACCORDING to a newspaper despatch several young Russian women who are students at the University of Turin are being kept under the surveillance of emissaries of the Russian secret service police because they are suspected of being engaged in attempting to discover the secret of the aqua tofana, the mysterious poison said to have been employed with such success by the Borgias. On the occasion of a rather serious illness suffered by the Czar some years ago, it was alleged that he had been poisoned by means of a pair of gloves impregnated with one of the old Italian poisons, the composition of which has been rediscovered by Russian revolutionaries by search among the old manuscripts in which the Biblioteca Nazionale at Turin is especially rich.—*Med. Age.*

---

THE Troup County Medical Society met in the rooms of the Elm City Club, May 10, 1906, and elected the following officers for the ensuing year: President, Dr. J. S. Horsley, Sr., West Point, Ga.; Vice-president, Dr. A. J. Tuggle, LaGrange, Ga.; Secretary and Treasurer, Dr. Henry W. Terrell, LaGrange, Ga. The resolutions of the House of Delegates of the Georgia Medical Association, relative to the fee of the Old Line Life Insurance examinations being fixed at five dollars, was unanimously adopted, as was also one endorsing the Hepburn national pure food bill, which has passed the House and is now before the Senate, and requesting Senators Bacon and Clay to support the same. Troup has one of the best organized medical societies in the State, as it has 28 members out of 32 licensed practitioners in the county.

---

THE ALUMNI ASSOCIATION OF THE MEDICAL DEPARTMENT OF THE UNIVERSITY OF GEORGIA.—At a meeting, held at Augusta, on Thursday, April 19th, this association was reorganized and the following officers were elected: President, Dr. J. Lawton Hiers, of Savannah; first vice-president, Dr. S. R. Gibson, of Thomson; second vice-president, Dr. W. W. Pilcher, of Warrenton; secretary and treasurer, Dr. C. W. Crane, of Augusta. The next meeting of the association will be in celebration of its seventy-fifth anniversary, and already there have been plans suggested for this occasion. The celebration will probably be the largest ever held by the alumni. Some of the most prominent physicians of the State have graduated at the college, and there will be a grand reunion of them on the seventy-fifth anniversary.

---

WHEN New York was a moderate-sized city, say thirty years ago, the veterans of medicine tell us that each prominent member of our profession was known by sight by all New Yorkers to the manor born. This was certainly true of Professor Ogden Doremus, who died a few days ago at a good old age of eighty and more. Dr. Doremus in his tall form, his luxurious growth of black hair, with his cloak thrown over his shoulders, and with his sonorous voice warmly greeting his friends, who comprised about all the community, hastening along Fourth avenue, between the Free Academy on Twenty-third street, and Bellevue College on Twenty-sixth street, was one of the features of scientific and medical New York. A more skilful chemical teacher, a more genial man, never lived. New York is now too large, too crowded, too shaded by sky-scrapers to miss anybody, or to remember many, but in the circle still existing of those who knew medical New York of the olden time, with Doctors Mott, Draper, Parker, Markoe, Post, Van Buren, Marion-Sims, Professor Doremus will be regretfully missed. A great figure has passed off the stage.—*The Post-Graduate, April 6.*

---

THE Widener Memorial Home for Crippled Children, at York road and Olney avenue, was opened on Saturday, March 3d. The institution was founded by Mr. Peter A. B. Widener as a memorial to his wife. Children suffering from deformities, and between the ages of six and sixteen years, will be received in the institution, where they will be taught truck farming, market gardening, poultry raising, horticulture, sewing, cooking, housework, dressmaking, millinery, stenography, telegraphy, carving, tailoring, basket-making, shoemaking, printing, silver engraving, draughting, designing, etc. The plan of the school includes a complete hospital department in which modern orthopedic methods will be employed for the correction of the deformities, after which the patients will be transferred to the industrial department, where their education will begin. The buildings, grounds, etc., will cost $2,000,000, and Mr. Widener has endowed the home with $3,000,000 for maintenance. Dr. De Forest Willard, who is in charge of the hospital department, Mayor Weaver and Col. A. K. McClure, made addresses at the dedicatory exercises.—*N. Y. Medical Journal, March 24, 1906.*

DR. E. D. RICHARDSON and Dr. J. W. Hart, county physicians, whose work at the convict camp has recently been criticized by the grand jury, have been fully exonerated by the board of Fulton County Commissioners. The following resolution was submitted:

"Whereas, The grand jury of Fulton county for the March term, 1906, in its presentments, reported that the county physicians, Dr. John W. Hurt and Dr. E. D. Richardson, had been guilty of gross neglect in their treatment of the convicts and recommended that the board of commissioners of roads and revenues discharge them from their positions; and,

"Whereas, Doctors Hurt and Richardson filed with said board of commissioners a written request for an investigation of the charges and their treatment of the convicts; and,

"Whereas, An exhaustive investigation of the charges and evidence taken in support of said charges and in refutation thereof has been heard by said board of commissioners.

Resolved, By the board of commissioners of roads and revenues of said county, That Doctors John W. Hurt and E. D. Richardson be, and they are, fully exonerated from said charges, and the same are dismissed."

---

### ST. JOSEPH'S INFIRMARY.

From a modest beginning, St. Joseph's Infirmary has, in the quarter of a century, grown in strength, equipments, and all those appointments which, with the recent discoveries in the medical world, have done so much to relieve the unfortunate and make possible, cures that were unknown a generation ago. In all the progress along aseptic, surgical and other lines, St. Joseph's Infirmary has kept step with the very foremost in the procession, and as a consequence, the opportunities now offered in caring for the sick at this institution are the pardonable pride not alone of the leading physicians of Atlanta, but of the entire State as well, and very many of these have improved these opportunities in caring for patients requiring just those conditions which are found at "St. Joseph's." The many improvements which have been made during the past twenty-five years, and especially of the past five or ten years, have been known to physicians throughout the State, and to many of the citizens as well. Among this number many have been treated for illness of various kinds, and the information which they have spread among their friends has been among the happiest and most valuable assets of the institution. The press of Atlanta and Georgia have also been ever ready to credit this Infirmary with the accomplishment of a mission which is of the greatest importance to the city and the commonwealth. But there can be but little doubt that it is the information which comes first hand, the testimony of those who have served a sentence imposed by disease, a sentence made shorter and far more pleasant by both the accomplished attendants and the perfect appointments that have made the name of "St. Joseph's" a veritable synonym of all that is kindliest, most comfortable and best among Atlantans, Georgians, and Southerners generally.

PERSONAL RELATIONS A FACTOR.—But St. Joseph's Infirmary is most fortunate in having more than these appointments and attendants which pertain to the professional side of such an institution alone. This is an indescribable something which suggests most forcibly and most pleasantly home influences and personal relations so seldom found in places where the public, and more particularly the suffering public, are always welcomed. In this way St. Joseph's Infirmary has ever disproved the old-time idea that hospitals were mere institutions for prisoners of pain, and substituted rather bright and cheery rooms for the comfort of the wayfarer destined to travel through the valley of suffering, and the kindly attention of those who are companionable, capable and competent. While directly under the supervision of the Sisters of Mercy, there is never, the least suggestion pertaining to secular influences, although the atmosphere and every surrounding is raised to a higher plane through the consecration of these Sisters, who have relinquished their every personal pleasure or ambition in a lifetime of self-sacrifice in the interest of the afflicted.

